# Validation of the Second Version of the LittlEARS^®^ Early Speech Production Questionnaire (LEESPQ) in Romanian-Speaking Children with Normal Hearing

**DOI:** 10.3390/audiolres15010009

**Published:** 2025-01-22

**Authors:** Alina-Catalina Ivanov, Luminita Radulescu, Sebastian Cozma, Madalina Georgescu, Bogdan Cobzeanu, Adriana Neagos, Petronela Moraru, Alma Maniu, Corina Butnaru

**Affiliations:** 1 ENT Clinic Department, “Grigore T. Popa” University of Medicine and Pharmacy Iasi, 700115 Iasi, Romania; alina-catalina.bordeianu-ivanov@d.umfiasi.ro (A.-C.I.);; 2 ENT Clinic Department, Carol Davila University of Medicine and Pharmacy Bucharest, 020021 Bucharest, Romania; 3 ENT Clinic Department, George Emil Palade University of Medicine, Pharmacy, Science and Technology of Targu Mures, 540142 Targu Mures, Romania; 4 ENT Clinic Department, Iuliu Hațieganu University of Medicine and Pharmacy Cluj, 400347 Cluj, Romania

**Keywords:** language development, Romanian language, auditory evaluation, LEESPQ, early verbal skills

## Abstract

**Objectives:** The objectives of the current study were to validate the LittlEARS^®^ Early Speech Production Questionnaire (LEESPQ) in Romanian and to evaluate the psychometric properties of the Romanian version of the questionnaire for Romanian children with normal hearing. The LEESPQ was created and tested for the assessment of preverbal and early verbal skills (0–18 months) in children with normal hearing. **Methods:** The English version of the LittlEARS^®^ Early Speech Production Questionnaire (LEESPQ) was adapted into Romanian language using a translation/back-translation procedure and validation of the content before applying the questionnaire. The Romanian version was applied to the parents of 232 children with normal hearing, aged between 0 and 18 months. The questionnaire was statistically analyzed to assess its reliability, internal consistency, predictive accuracy, and the influence of gender on children’s scores. **Results:** Statistical analyses confirmed the LEESPQ’s reliability (α = 0.876) and high predictive accuracy (λ = 0.951). Age correlated strongly with total scores (ρ = 0.67; p < 0.001), supporting the age-dependent progression of speech production milestones. Gender did not significantly affect the scores. Normative curves and minimum expected scores were established for each age group. **Conclusions:** This study confirmed that the Romanian version of the LEESPQ is a reliable, valid, language-independent instrument, useful in the assessment of language development in children with normal hearing, aged up to 18 months.

## 1. Introduction

Language acquisition begins at birth and progresses rapidly in the first 18 months of life, encompassing receptive and expressive language skills. Expressive language, the ability to convey ideas, thoughts, and emotions, develops alongside receptive understanding. By 18 months, children typically produce dozens of words as part of their emerging vocabulary.

Newborn hearing screening and early intervention for children with congenital hearing loss have created a demand for tools to assess hearing development in infants (0–18 months). Along with the objective measurements of hearing in infants and small children, the subjective measurements are equally important, thus helping to obtain information related to the state of hearing in these age categories [1].

The LittlEARS^®^ test battery was designed by multidisciplinary experts to track the auditory and verbal development of children. One part of this test battery is the LittlEARS^®^ Auditory Questionnaire (LEAQ), created by Coninx and colleagues, which evaluates auditory development in infants aged 0 to 24 months [2].

Another part of the LittlEARS^®^ test battery is the LittlEARS^®^ Early Speech Production Questionnaire (LEESPQ) which evaluates the early development of speech and language in infants aged 0–18 months [3]. It tracks critical stages such as reflexive sounds, canonical babbling, word–object association, and first-word production.

The LEESPQ is a comprehensive questionnaire consisting of 27 age-dependent questions designed to monitor crying, prelingual emotions, reflexive behavior, pre-canonical vocalizations, canonical, and post-canonical vocalizations in children up to age of 18 months [4]. The stages of speech production are targeted: the production of reflexive sounds, the production of adult-like vowels, the second phase of babbling, word-like utterances, sound–object association, and the production of the first words [4].

The LEESPQ includes questions designed to assess key aspects of speech development in infants and young children. Examples of these questions include the following: “Does your child make happy sounds? (e.g., while playing, while taking a bath)”, “Does your child make vowel sounds? (e.g., ‘ah’, ‘eh’, ‘ee’, ‘oh’)”, and “Does your child make a range of consonant sounds? (e.g., m, b, p, w, h, g)”. These questions are structured to capture various stages of early speech production, providing valuable insights into the child’s verbal development.

In 2022, approximately 9% of the individuals in Romania with peripheral hearing disorders were children [5]. For every 1000 live births, three of the newborns have hearing impairments, so hearing loss is a congenital condition with a high incidence [5]. Diagnosis as early as possible and early intervention, including hearing care and the provision of hearing aids, are essential for auditory and verbal development during the critical initial period of a child’s life. The conceptual processing of communication is represented by receptive language (understanding) and expressive language (the ability to transmit information, feelings, ideas, thoughts) [6]. The functional language of the child develops in the first three years as part of its general development, based on the components of non-verbal communication and on the progressive learning of lexical, syntactic, phonological capacity, both on the receptive side and on the expressive side [7]. Studies show that around the age of 18 months, children have an active vocabulary of dozens of words [8].

The LEESPQ was initially developed and validated in German [9]. Due to the cultural differences between different languages, the authors recommended the translation and validation of the questionnaire in each new language [9]. Thus, the LEESPQ was validated in German on 362 children with normal hearing [9], in Turkish on 222 children with normal hearing [10], in Arabic on 198 children with normal hearing [11], and in Dutch for 355 children with normal hearing [12].

The usefulness of a self-administered subjective instrument for monitoring the stages of auditory and verbal development is important and helps to identify hearing loss in children in the first stage of life [13]. Structured questionnaires applied to parents are useful tools in the speech evaluation of small children. Parents/guardians can complete the LEESPQ after observing the child’s natural behavior in a familiar setting [3]. Most of the time, in the clinical setting, children’s preverbal auditory behaviors cannot be observed. Some of the children are too young to participate in hearing tests, while other children do not cooperate in unfamiliar environments [14,15,16]. Thus, by completing the questionnaire, parents/guardians can describe the children’s auditory responses in real situations. The questionnaire is non-invasive for the child, convenient for the parent/guardian, and even if parents are prone to overestimate the children’s abilities, the questionnaire is still a diagnostic tool useful in evaluating the communication skills of infants and young children [9].

## 2. Materials and Methods

### 2.1. Translation

The LittlEARS^®^ Early Speech Production Questionnaire (LEESPQ) was initially developed in German. The version, later translated into English, was the basis for the adaptation of the questionnaire in other languages. The English version was adapted into Romanian using the translation/back translation procedure recommended by the International Testing Commission (ITC) [17]. The goal was to obtain a linguistically correct version [13]. According to the ITC Guide, the adaptation of the questionnaire in another language is a rigorous process, which considers both linguistic, cultural, and psychometric aspects. The new model must ensure that the items of the translated version have the same relationship with the variable as in the original questionnaire. In the translation/retranslation design, three stages are completed: (a) translation of the original version into the target language; (b) the first translation is later translated into the original language; (c) the two versions of the test in the original language (the first version and the version translated into the target language) are compared to evaluate the quality of the translation [13]. The comparison between the original and back-translated versions of the questionnaire was performed by an expert who carefully reviewed both versions to assess linguistic accuracy, cultural alignment, and consistency in meaning.

### 2.2. Subjects

Subjects were recruited through two primary channels: some were enrolled via the national newborn hearing screening program, while others were recruited during routine visits to the hospital, where parents accompanied their children for various medical conditions, unrelated to hearing. In the latter case, these parents were informed about the study and voluntarily agreed to participate.

The subjects of this cross-sectional study were a number of 232 parents of children with normal hearing, aged between 0 and 18 months. Out of 232 subjects, 121 (52.2%) were girls, 106 (45.7%) boys, and 5 with undeclared gender. The children’s inclusion criteria were the absence of known disabilities (hearing loss, neurological disorders, prematurity), as well as the lack of a significant otological and/or medical history, or exposure to any high-risk factor in the pre/peri/postnatal period [18]. All children were examined using the OAE before the parent completed the questionnaire. Children who did not pass the test were excluded from the study and referred to the audiology service. The number of children per age group is shown in Table 1.

### 2.3. Materials

The LittlEARS^®^ Early Speech Production Questionnaire (LEESPQ) was applied to the parent/legal guardian, by three different interviewers from three distinct regions of the country. Each interviewer carefully explained how to complete the questionnaire without influencing the process in any way. The questionnaire contains 27 yes/no questions that evaluate the reflexive behavior, crying, prelingual emotions, and language of infants aged between 0 and 18 months. The total score that can be obtained is 27 points. After obtaining consent, the parents/legal guardians were instructed on how to complete the questionnaire, answering “Yes” or “No” to the questions asked. Thus, if they observed a behavior described in the question, they answered “Yes”, and if they did not observe that behavior in the child, they answered “No”. “Yes” answers receive one point, while “No” answers receive zero points.

### 2.4. Administration

To maintain homogeneity among the participants, all parents/guardians were Romanian speakers, and the selected children had a similar auditory exposure. If the parents/guardians observed a certain behavior mentioned in the question, they were asked to answer “Yes” and “No” for the items in the test that were not observed in their child.

### 2.5. Ethics

All participants were volunteers and received no compensation for their participation in the study. The confidentiality of the personal data of the participants was preserved. Before accessing the medical files and applying the questionnaire, the approval of the Research Ethics Commission of the University of Medicine and Pharmacy “Gr. T. Popa Iasi” was obtained.

All parents/guardians of the children were informed about the necessity, purpose, and objectives of the study, as well as the procedures involved in data collection. Informed consent for participating in the study was obtained from them. All procedures followed in the study involved the application of non-invasive methods for the participant.

### 2.6. Validation Procedure

To validate the questionnaire, various measures were evaluated, including the relationship between the overall score and the children’s ages (using Pearson’s correlation coefficient), the consistency within the scale itself (measured by Cronbach’s alpha), the reliability of dividing the scale into two parts (assessed through the Spearman–Brown split-half coefficient), and the scale’s ability to predict outcomes accurately (determined by Guttman’s lambda).

To assess the questionnaire’s capability to gauge age-related speech production ability, Pearson’s correlation coefficient (r) was calculated to determine the relationship between age and the individual questionnaire items. Furthermore, the impact of the child’s gender on the results was examined.

## 3. Results

### 3.1. Statistical Analysis

Cronbach’s alpha was used to assess the internal consistency and reliability of the scale. A value of α = 0.876 was obtained, signifying good internal consistency.

The full test reliability of the questionnaire was estimated using the Spearman–Brown split-half coefficient. The estimate of the full test reliability of questionnaire was r = 0.694, which indicates an acceptable reliability.

The correlation between age and total score was determined to obtain information about the ability of the questionnaire to evaluate age-dependent speech production behavior. A correlation coefficient of r = 0.67 was found, which shows that older children are more likely to obtain higher scores.

The predictive accuracy of the scale was assessed using Guttman’s Lambda (λ), a measure that evaluates how precisely the dependent variable (total score) can be forecasted from the independent variable (age). A value of λ = 0.951 was computed for this study, suggesting a very high predictability.

There is no significant difference between female children (n = 106, mean = 16.25, SD = 5.5) and male children (n = 121, mean = 17.02, SD = 5.22) scores.

An ANOVA revealed that gender had no significant impact on the total score.

**Figure 1:**
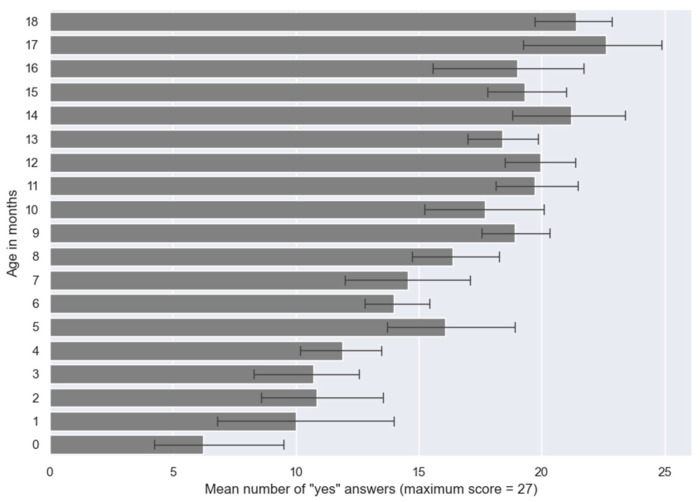
Mean number “yes” answers (± standard deviation) per age.

Older children were more likely to answer ‘yes’ than younger ones, as illustrated in Figure 1. The strong and statistically significant correlation between age and overall scores (r = 0.67; *p* < 0.001) underscores the tight linkage between the development of children’s speech production capabilities and their age.

### 3.2. Norm Curve

**Figure 2:**
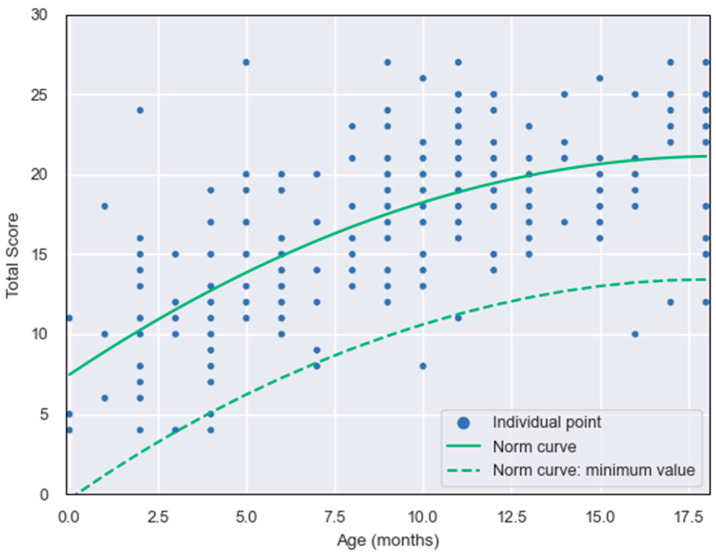
Regression curve (quadratic) with age as the independent and total score as the dependent variables and the lower bound of the confidence interval (5–95%). The dots indicate observed values.

A regression analysis was conducted to create a normative curve for early speech production in children aged 0 to 2 years. In this analysis, “age” served as the independent variable and “total score” as the dependent variable. The least squares method was used for calculations. The chosen model is a second-order polynomial regression, represented by this Equation:

y = −0.04x^2^ + 1.48x + 7.47

where x = age and y = total score (F = 107.7, df = 229, *p* < 0.001). The coefficient for the model showed that 48.5% of the variance in the total scores can be explained by age. A scatter plot of the raw data and the generated norm curve is shown in Figure 2.

The results from the regression analysis were used to identify the confidence intervals where there is a 95% probability of finding the age-specific values. The lower edge of the confidence interval was established as the minimum threshold for children with normal hearing. Scores surpassing this threshold are viewed as aligning with healthy, age-suitable speech production development abilities.

Table 2 shows the Pearson correlation between the probability of positive response (“yes”) and age for each item. Several items demonstrated strong positive correlations (ρ > 0.5), indicating a robust age dependency:

Question 20 (r = 0.65): “Does your child imitate animal noises or the noises of vehicles?”

Making sounds mimicking animals or vehicles demonstrates growing symbolic and imitation skills as age advances, a key milestone in speech development.

Question 21 (r = 0.58): “Does your child give ‘made-up’ names to objects, animals, or people?”

This reflects the development of symbolic and imitation skills, critical milestones in early speech and cognitive development. As children grow, they increasingly mimic environmental sounds, which aids in lexical acquisition. The literature suggests that the ability to produce such imitative sounds correlates with improved phonological and vocabulary skills [13].

Several items exhibited moderate or weak correlations with age, suggesting varied developmental patterns:

Questions 1 and 3 (r = −0.17 and r = −0.19): Crying or lip movement unrelated to feeding may be less age-specific and more dependent on individual temperament or environmental factors.

### 3.3. Calculation of Critical Values/Minimum Values

The minimum values, identified as the critical lower limit (the lower boundary of the confidence interval), necessary for a child with normal hearing to attain development consistent with age-appropriate speech production are shown in Table 3. Children with scores below this lower limit have a small probability (less than 5%) of exhibiting age-appropriate speech production development abilities.

## 4. Discussion

This study aimed to evaluate the validity of the Romanian version of the LittlEARS^®^ Early Speech Production Questionnaire (LEESPQ) and its applicability as a tool for assessing speech development in Romanian children with normal hearing, aged 0–18 months.

The LEESPQ was first translated into Romanian, then back-translated into English to ensure accuracy. It was subsequently refined and culturally adapted to align with the nuances of the Romanian language.

The findings of this study strongly support the validity of the Romanian LEESPQ, which is consistent with the results of validations in Turkish, and Arabic. These comparisons underscore the robustness of the LEESPQ as a cross-cultural instrument for assessing early speech production.

The Romanian LEESPQ demonstrated excellent internal consistency (Cronbach’s α = 0.876), comparable to the Turkish (α = 0.940) and Arabic (α = 0.861) versions, underscoring its reliability in assessing speech production exclusively.

This consistency validates the LEESPQ’s utility in identifying subtle variations in speech production development among children across cultures. Predictive accuracy, as indicated by Guttman’s Lambda (λ = 0.694) for the Romanian version, is similarly corroborated by the Turkish (λ = 0.910) and Arabic (λ = 0.533) versions, confirming the tool’s efficacy in predicting developmental trajectories with age.

Gender neutrality, a critical aspect of the LEESPQ, was affirmed in the Romanian context, with no significant differences observed in scores between boys and girls. This mirrors the findings in the Turkish (*p* = 0.349) and Arabic validations, which also reported no gender-based differences in scoring.

In this study, a strong correlation was observed between the child’s age and the total score (r = 0.67), supporting the age-dependent nature of speech development. This finding aligns with the Turkish version (r = 0.850) and Arabic version (r = 0.518), which also demonstrated a significant age dependency in scoring. The Romanian findings reinforce the premise that older children exhibit greater mastery of early speech production milestones, as reflected in progressively higher scores.

Clinically, the regression curve can serve as a valuable tool for benchmarking normal developmental progress. It provides healthcare professionals with a visual framework for identifying deviations from typical speech production patterns. For example, children whose scores fall below the lower confidence limit may warrant further evaluation for potential speech delays or auditory issues.

The strong correlations observed for several items of the LEESPQ validate their use as reliable markers for age-appropriate speech development. Clinicians can use these items to track developmental milestones—high-correlation items like Questions 16, 20, and 23 provide benchmarks for assessing typical progress. They also can identify atypical patterns—items with weak or negative correlations may highlight areas for further investigation, especially if responses deviate significantly from normative expectations. By identifying delays early, clinicians can implement targeted interventions to support speech and language development.

By integrating findings from the Turkish and Arabic validations, this study reinforces the LEESPQ’s cross-cultural applicability and universality as a tool for early auditory and speech development assessment. The comparability of psychometric properties across these validations demonstrates that the LEESPQ transcends linguistic and cultural barriers while maintaining its effectiveness.

In conclusion, the Romanian LEESPQ emerges as a reliable, valid, and culturally adaptable instrument, strengthening the body of evidence supporting the LEESPQ’s role as a global standard for early speech production assessment. It provides a vital resource for clinicians, researchers, and policymakers, enabling early identification of developmental delays and guiding timely interventions. Future studies could explore its utility in diverse populations, including children with hearing impairments, to further validate its broad-spectrum applicability and refine its diagnostic capabilities.

## 5. Conclusions

The results of this study confirm that the Romanian version of the LittlEARS^®^ Early Speech Production Questionnaire (LEESPQ) is a valid and reliable tool for assessing speech development in children aged 0–18 months. By demonstrating strong psychometric properties and a robust correlation between age and scores, this instrument proves to be a valuable resource in both clinical and research settings for evaluating developmental milestones in children with normal hearing.

Looking ahead, the LEESPQ holds significant potential for further applications and developments. Future research will explore its utility in monitoring auditory and speech development in children with hearing impairments, including those with cochlear implants or who undergo early intervention programs, such as hearing-aid fitting. Additionally, its adaptability across diverse linguistic and cultural contexts invites further validation studies in other populations, enabling its use as a global standard for early speech production assessment.

To enhance its diagnostic capabilities, we also aim to integrate the LEESPQ into broader developmental monitoring frameworks, potentially incorporating digital platforms to facilitate real-time data collection and analysis. These advancements could provide clinicians and researchers with deeper insights into individual developmental trajectories, supporting earlier and more targeted interventions. Ultimately, the continued refinement and application of the LEESPQ will help ensure that children with hearing or speech delays receive timely and effective care, fostering better long-term outcomes.

## Figures and Tables

**Table 1:** Number of children per age group. The completed month defines the previous age category.

Age (Months)	n	Age (Months)	n
0–1	4	>9–10	22
>1–2	5	>10–11	13
>2–3	15	>11–12	17
>3–4	7	>12–13	16
>4–5	21	>13–14	13
>5–6	11	>14–15	5
>6–7	16	>15–16	10
>7–8	11	>16–17	7
>8–9	11	>17–18	28

**Table 2:** Pearson correlation (r) between age and the probability of a positive response (“yes”) to an item.

Item No.	(r)	Item No.	(r)
q1	−0.17	q14	0.33
q2	0.17	q15	0.09
q3	−0.19	q16	0.62
q4	−0.08	q17	0.24
q5	0.15	q18	0.53
q6	−0.07	q19	0.26
q7	0.31	q20	0.65
q8	0.31	q21	0.58
q9	0.3	q22	0.51
q10	0.29	q23	0.62
q11	0.38	q24	0.54
q12	0.45	q25	0.42
q13	0.31	q26	0.28
q14	0.33	q27	0.45

**Table 3:** Expected mean values and minimum mean values of age dependent speech production ability.

Age (Months)	Expected Values	Minimum Values	Age (Months)	Expected Values	Minimum Values
0–1	7	0	>9–10	18	10
>1–2	9	1	>10–11	18	11
>2–3	10	3	>11–12	19	11
>3–4	12	4	>12–13	19	12
>4–5	13	5	>13–14	20	12
>5–6	14	6	>14–15	20	13
>6–7	15	7	>15–16	21	13
>7–8	16	8	>16–17	21	13
>8–9	17	9	>17–18	21	13

## Data Availability

Data will be made available on request.
